# Predicting survival of patients with idiopathic pulmonary fibrosis using GAP score: a nationwide cohort study

**DOI:** 10.1186/s12931-016-0454-0

**Published:** 2016-10-18

**Authors:** Sang Hoon Lee, Song Yee Kim, Dong Soon Kim, Young Whan Kim, Man Pyo Chung, Soo Taek Uh, Choon Sik Park, Sung Hwan Jeong, Yong Bum Park, Hong Lyeol Lee, Jong Wook Shin, Eun Joo Lee, Jin Hwa Lee, Yangin Jegal, Hyun Kyung Lee, Yong Hyun Kim, Jin Woo Song, Sung Woo Park, Moo Suk Park

**Affiliations:** 1 Department of Internal Medicine, Seoul National University College of Medicine, Division of Pulmonary and Critical Care Medicine, Seoul National University Bundang Hospital, 166, Gumi-ro, Bundang-gu, 463-707 Seongnam-si, Gyeonggi-do Republic of Korea; 2 Division of Pulmonology, Department of Internal Medicine, Severance Hospital, Institute of Chest Diseases, Yonsei University College of Medicine, 50-1, Yonsei-ro, Seodaemun-gu, Seoul, 120-752 South Korea; 3 Division of Pulmonary and Critical Care Medicine, University of Ulsan College of Medicine, Asan Medical Center, Seoul, South Korea; 4 Division of Pulmonary and Critical Care Medicine, Department of Internal Medicine and Lung Institute, Seoul National University College of Medicine, Seoul, South Korea; 5 Division of Pulmonary and Critical Care Medicine, Samsung Medical Center, Sungkyunkwan University School of Medicine, Seoul, South Korea; 6 Division of Allergy and Respiratory Medicine, Department of Internal Medicine, Soonchunhyang University Seoul Hospital, Seoul, South Korea; 7 Division of Allergy and Respiratory Medicine, Department of Internal Medicine, Soonchunhyang University Bucheon Hospital, Seoul, South Korea; 8 Division of Pulmonology, Department of Internal Medicine, Gachon University Gil Medical Center, Incheon, South Korea; 9 Division of Pulmonary, Allergy & Critical Care Medicine, Department of Internal Medicine, Hallym University Kangdong Sacred Heart Hospital, Chuncheon, South Korea; 10 Pulmonary Division, Department of Internal Medicine, Inha University Hospital, Incheon, South Korea; 11 Division of Pulmonary Medicine, Department of Internal medicine, Chung Ang University College of Medicine, Seoul, South Korea; 12 Division of Respiratory and Critical Care Medicine, Department of Internal Medicine, Korea University Anam Hospital, Korea University College of Medicine, Seoul, South Korea; 13 Department of Internal Medicine, Ewha Womans University School of Medicine, Ewha Medical Research Institute, Seoul, South Korea; 14 Division of Pulmonary Medicine, Department of Internal Medicine, Ulsan University Hospital, University of Ulsan College of Medicine, Ulsan, South Korea; 15 Division of Critical Care and Pulmonary Medicine, Department of Internal Medicine, Inje University Pusan Paik Hospital, Gimhae, South Korea; 16 Division of Allergy and Pulmonology, Department of Internal Medicine, Bucheon St. Mary’s Hospital, The Catholic University of Korea School of Medicine, Seoul, South Korea

**Keywords:** Idiopathic pulmonary fibrosis, GAP stage, Prognosis

## Abstract

**Background:**

The clinical course of idiopathic pulmonary fibrosis (IPF) varies widely. Although the GAP model is useful for predicting mortality, survivals have not yet been validated for each GAP score. We aimed to elucidate how prognosis is related to GAP score and GAP stage in IPF patients.

**Methods:**

The Korean Interstitial Lung Disease Study Group conducted a national survey to evaluate various characteristics in IPF patients from 2003 to 2007. Patients were diagnosed according to the 2002 criteria of the ATS/ERS. We enrolled 1,685 patients with IPF; 1,262 had undergone DL_CO_ measurement. Patients were stratified based on GAP score (0–7): GAP score Group 0 (*n* = 26), Group 1 (*n* = 150), Group 2 (*n* = 208), Group 3 (*n* = 376), Group 4 (*n* = 317), Group 5 (*n* = 138), Group 6 (*n* = 39), and Group 7 (*n* = 8).

**Results:**

Higher GAP score and GAP stage were associated with a poorer prognosis (*p* < 0.001, respectively). Survival time in Group 3 was lower than those in Groups 1 and 2 (*p* = 0.043 and *p* = 0.039, respectively), and higher than those in groups 4, 5, and 6 (*p* = 0.043, *p* = 0.032, and *p* = 0.003, respectively). Gender, age, and DL_CO_ (%) differed significantly between Groups 2 and 3. All four variables in the GAP model differed significantly between Groups 3 and 4.

**Conclusion:**

The GAP system showed significant predictive ability for mortality in IPF patients. However, prognosis in IPF patients with a GAP score of 3 were significantly different from those in the other stage I groups and stage II groups of Asian patients.

**Electronic supplementary material:**

The online version of this article (doi:10.1186/s12931-016-0454-0) contains supplementary material, which is available to authorized users.

## Background

Idiopathic pulmonary fibrosis (IPF) is a specific form of diffuse interstitial lung disease (DILD) that mainly occurs in adults over the age of 50 [1]. It is a chronic, progressive, irreversible, fibrosing interstitial pneumonia, characterized by limited to the lungs [2]. While the etiology of IPF is unknown, it is related to a histological and/or radiological “usual interstitial pneumonia” (UIP) pattern [1]. Morbidity and mortality are high in IPF—the median survival time is only 2.5 to 3.5 years—and the clinical course and prognosis vary widely among individual patients [3]. This high variability makes predicting prognosis difficult, which in turn causes problems with treatment planning. Therefore, physicians must be better equipped to predict the clinical course of IPF if they are to provide precise prognoses and adequate treatment to patients.

Previous studies have shown that age, gender, lung function change, radiological pattern, histological variability, dyspnoea, cough, pulmonary artery hypertension, amount of elastic fiber, and some molecular biomarkers are associated with prognosis [4–10]. Some investigators have attempted to predict clinical course using these prognostic factors [11]. However, none of these predictive models have been widely adopted, as they are difficult to use or lack external validation. In 2012, Ley et al. suggested a novel system for staging IPF that is similar to those used in asthma, chronic obstructive pulmonary disease (COPD), and lung cancer [12]. The so-called GAP index and staging system uses of four variables: gender (G), age (A), and two pulmonary physiological parameters (P)—percentage predicted forced vital capacity (FVC [%]), and percentage predicted diffusion capacity of the lungs for carbon monoxide (DL_CO_ [%]). These four variables are commonly measured at the initial visit and are easily followed up. This system has helped clinicians to predict prognosis and decide on management strategies. Although this GAP model is simple-to-use for predicting mortality, prognoses have not yet been evaluated for each GAP score. The purpose of our study was to validate, using national survey data, how prognosis is related to GAP score and GAP stage in patients with IPF.

## Methods

### Patient selection

The study involved patients who had been diagnosed with idiopathic interstitial pneumonia (IIP) at 54 university and teaching hospitals between January 1, 2003 and December 31, 2007. At each hospital, pulmonary specialists (pulmonologists, chest radiologists, and pathologists) had confirmed the diagnoses, and data were reviewed by the Scientific Committee at the Korean Academy of Tuberculosis and Respiratory Diseases. IPF was diagnosed on the basis of the 2002 criteria of the American Thoracic Society/European Respiratory Society (ATS/ERS) [13]. Initially, we excluded patients who had a history of connective tissue disease, pneumoconiosis, or ingestion of either a cytotoxic agent or amiodarone, and all of which are well-known to cause interstitial lung disease. Additionally, we excluded patients with suspected chronic hypersensitivity pneumonitis; such decisions were made on the basis of history, laboratory data, and committee conference.

**Fig. 1:**
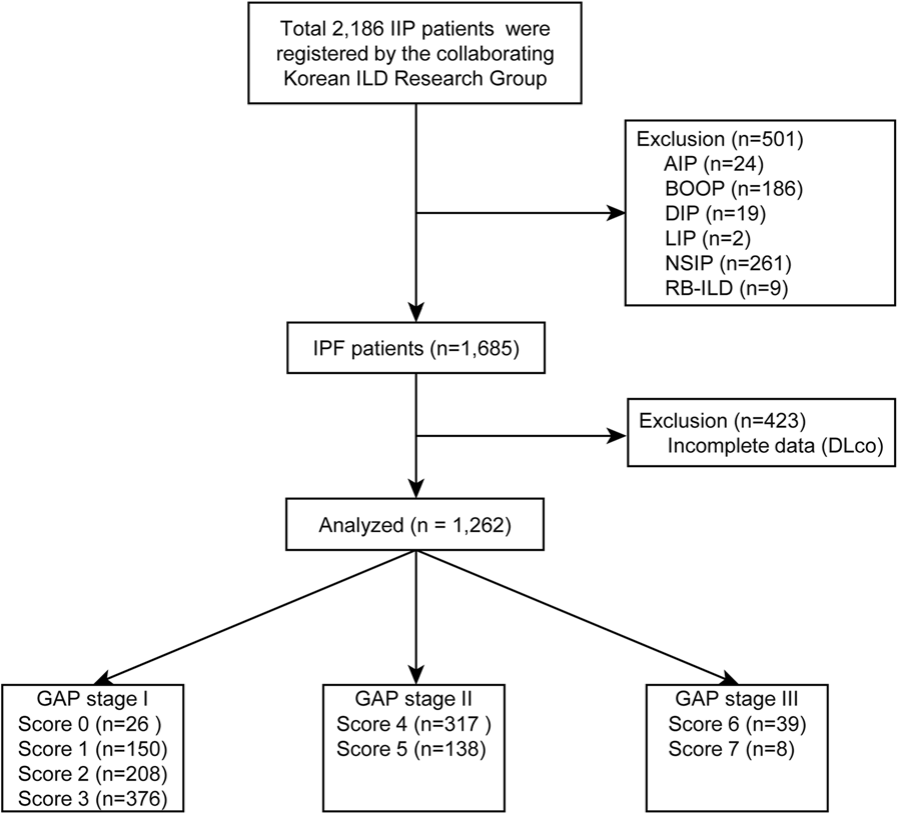
Flow chart showing inclusion and exclusion of patients in the study. A total of 1262 IPF patients were analysed in this study, excluding 501 with other interstitial lung disease and 423 who had not undergone pulmonary function testing that had included DL_CO_. Note: Groups with a total GAP score of 0 and 7 were excluded because they contained too few patients and because the baseline characteristics of patients with GAP score 0 were significantly different (all women, never smokers). No patients with a GAP score of 8 were included, because the “unable to perform” category in DL_CO_ was not checked in this study. Definition of abbreviations: IIP, idiopathic interstitial pneumonia; ILD, interstitial lung disease; AIP, acute interstitial pneumonia; BOOP, bronchiolitis obliterans organizing pneumonia; DIP, desquamative interstitial pneumonia; LIP, lymphocytic interstitial pneumonia; NSIP, non-specific interstitial pneumonia; RB-ILD, respiratory bronchiolitis-associated interstitial lung disease

In total, 2,186 patients with idiopathic interstitial pneumonia (IIP) were registered; of these, patients with other forms of ILD than IPF (*n* = 501) were excluded from the study, as were patients who had not undergone pulmonary function testing (PFT) that included DL_CO_ measurement (*n* = 423). Ultimately, 1,262 patients were included in the study: 760 at GAP stage I, 455 at stage II, and 47 at stage III (Fig. 1). We reviewed the clinical, radiological, and physiological data of all the included patients. With regard to physiological data, we investigated FVC, FVC (%), forced expiratory volume in one second (FEV_1_), percentage predicted FEV_1_ (FEV_1_ [%]), total lung capacity (TLC), percentage predicted TLC (TLC [%]), DL_CO_, and percentage predicted DL_CO_ (DL_CO_ [%]). In addition, we evaluated patients’ C-reactive protein (CRP) levels, and examined their blood for the positivity of antinuclear antibody (ANA) and rheumatoid factor (RF). The composite physiologic index (CPI), which is a predictive model for IPF prognosis, was calculated as Well et al. reported [14]. All hospital data were entered into the ILD web-based registry (http://www.ild.or.kr/).

### GAP model

Total GAP score was calculated using the method suggested by Ley *et al* [12] (Table 1). All four clinical variables were examined: gender (woman: 0 points, man: 1 point), age (0–2 points), FVC (%) (0–2 points), and DL_CO_ (%) (0–3 points). We then divided the patients on the basis of GAP score (Groups 0–7): Group 0 (*n* = 26), Group 1 (*n* = 150), Group 2 (*n* = 208), Group 3 (*n* = 376), Group 4 (*n* = 317), Group 5 (*n* = 138), Group 6 (*n* = 39), and Group 7 (*n* = 8). In the physiological category, the “cannot perform” classification (3 points) of DL_CO_ measurement had not been recorded in the data used. For this reason, the total GAP score of 8 was not investigated in the current study. Additionally, we excluded patients with total GAP scores of 0 (*n* = 26), and 7 (*n* = 8), as these two groups contained much fewer patients than the other groups. The characteristics in Group 0, which contained only women who had never smoked, were significantly different from those in the other groups.

**Table 1:** GAP index and number (%) of patients

Variables	GAP Points	No. of patients
Gender		
Female	0	315 (25.7)
Male	1	913 (74.3)
Age, yr		
≤60	0	263 (21.4)
61–65	1	208 (16.9)
>65	2	757 (61.6)
Physiology		
FVC, % predicted		
>75	0	626 (50.9)
50–75	1	540 (44.0)
<50	2	62 (5.0)
DL_CO_, % predicted		
>55	0	735 (59.9)
36–55	1	399 (32.5)
≤35	2	94 (7.6)
Can not perform	3	-
GAP stage		
Stage I	0–3	760 (60.2)
Stage II	4–5	455 (36.1)
Stage III	6–8	47 (3.7)

Note: Values in parentheses are percentages

*GAP* gender, age, and 2 lung physiology variables (FVC and DL_CO_)

### Statistical analysis

Information was obtained from web-based questionnaires and medical records; it was stored and analysed using the Excel™ computer program. Analysis of variance (ANOVA) was used to compare continuous variables, and Bonferroni’s correction was used for *post-hoc* analysis. Pearson’s chi-squared test or Fisher’s exact test were used to compare categorical variables. Continuous variables were presented as mean ± standard deviation, or proportions within each group as a percentage.

To compare the GAP score groups in terms of survival times, Kaplan-Meier survival analysis and the log-rank test were used. In addition, multivariate analysis was conducted with Cox proportional hazard model. C-statistic was also performed for the GAP model at 1-year, 2-year, and 3-year. When performing the survival analysis, we censored the following conditions: (1) still alive at last visit (at last visit date), (2) lost to follow-up loss and (3) had undergone lung transplantation (at surgery date). Statistics were analysed using SPSS™ Version 20 (SPSS, Chicago, IL, USA). An adjusted p-value less than 0.05 was regarded as statistically significant.

## Results

### Demographic characteristics

There were 1,228 patients with a GAP score from 1 to 6. The baseline characteristics of these patients are summarized in Table 2. The mean age of the study population was 67.5 ± 9.3 years and was lowest in Group 1. The highest proportion of men occurred in Group 6 (*p* < 0.001). Although the patients in Group 1 had experienced the longest duration of respiratory symptoms at diagnosis, and those in Group 6 had experienced the shortest, this was not statistically significant (*p* = 0.133). With regard to smoking status, 83.3 % of patients in Group 6 were ever-smokers; the equivalent values in Groups 1 and 2 were 58.7 and 50.5 %, respectively. Furthermore, smoking duration and amount were higher in Group 6 than in the other score groups (*p* < 0.001 and *p* = 0.024, respectively). Patients with a higher GAP score tended to have been diagnosed using the clinical method rather than surgical lung biopsy. Specifically, the proportion of clinically diagnosed patients was 87.2 % in Group 6, whereas it was 22.0 % Group 1. The percentages of ANA and RF positivity did not differ significantly among the groups (*p* = 0.580 and *p* = 0.177, respectively). Increased CRP level was significantly associated with higher GAP score (*p* < 0.001). CPI also tended to increase as GAP score increased (*p* < 0.001). The mean value of CPI was significantly different between Group 3 and Group 4, although there was no significant difference between Group 2 and Group 3 after Bonferroni’s correction. The mean follow-up duration of the study population was 19.0 ± 16.0 months.

**Table 2:** Baseline characteristics of study population according to total GAP score

Variable	Total GAP score (*n* = 1,228)						*p*-value
	1 (*n* = 150)	2 (*n* = 208)	3 (*n* = 376)	4 (*n* = 317)	5 (*n* = 138)	6 (*n* = 39)	
Age, yr	56.2 ± 5.7	62.5 ± 8.9	69.0 ± 7.8	71.7 ± 7.6	72.4 ± 7.5	71.8 ± 5.9	<0.001^a^
Sex, male (%)	91 (60.7)	114 (54.8)	281 (74.7)	269 (84.9)	123 (89.1)	35 (89.7)	<0.001
Duration of symptoms at diagnosis (Month)	15.9 ± 27.9	9.8 ± 15.0	10.7 ± 20.6	10.8 ± 21.8	9.5 ± 17.8	5.4 ± 12.8	0.133
Smoking							<0.001
Non-smoker	57 (41.3)	98 (49.5)	106 (31.7)	80 (28.3)	33 (27.3)	6 (16.7)	
Former	36 (26.1)	49 (24.7)	139 (41.6)	126 (44.5)	59 (48.8)	19 (52.8)	
Current	45 (32.6)	51 (25.8)	89 (26.6)	77 (27.2)	29 (24.0)	11 (30.6)	
Smoking duration (yrs)	29.1 ± 9.1	29.8 ± 11.0	37.6 ± 11.6	38.0 ± 13.0	37.4 ± 15.7	41.3 ± 9.0	<0.001^a^
Smoking amounts (PYrs)	32.1 ± 18.2	30.7 ± 19.5	37.0 ± 18.0	36.6 ± 20.1	38.6 ± 25.7	40.0 ± 17.9	0.024^a^
Diagnostic method (%)							<0.001
Clinical	33 (22.0)	86 (41.3)	235 (62.5)	244 (77.0)	119 (86.2)	34 (87.2)	
Surgical	117 (78.0)	122 (58.7)	141 (37.5)	73 (23.0)	19 (13.8)	5 (12.8)	
Outcome							<0.001
Alive	92 (61.3)	118 (56.7)	167 (44.4)	114 (36.0)	36 (26.1)	10 (25.6)	
Dead	24 (16.0)	31 (14.9)	82 (21.8)	83 (26.2)	35 (25.4)	15 (38.5)	
Loss	34 (22.7)	59 (28.4)	127 (33.8)	120 (37.9)	67 (48.6)	14 (35.9)	
ANA positivity	34 (33.7)	31 (28.4)	63 (37.3)	38 (29.9)	20 (35.1)	4 (23.5)	0.580
RF positivity	21 (20.4)	18 (16.5)	42 (24.1)	36 (28.3)	14 (29.2)	2 (11.1)	0.177
CRP (mg/L)	1.3 ± 3.4	2.2 ± 4.3	4.0 ± 11.5	5.9 ± 15.9	7.6 ± 22.1	14.2 ± 38.7	<0.001^a^
CPI	28.0 ± 10.8	34.9 ± 12.3	35.8 ± 14.9	42.8 ± 12.8	55.7 ± 8.0	62.9 ± 6.8	<0.001^a^

Note: Values in parentheses are percentages.

CPI = 91.0 – (0.65 ^a^percent predicted DL_CO_) – (0.53 ^a^percent predicted FVC) + (0.34 ^a^percentage predicted FEV_1_)

*GAP* gender, age, and 2 lung physiology variables (FVC and DL_CO_), *ANA* antinuclear antibody, *RF* rheumatoid factor, *CPI* composite physiologic score

^a^the following post hoc comparisons were significant at the *p* = 0.05 level; all other comparisons were non-significant: Score 1 group versus Score 2, 3, 4, 5, 6 groups, Score 2 group versus Score 3, 4, 5, 6 groups, and Score 3 group versus Score 4, 5 groups (age); Score 1 group versus Score 3, 4, 5, 6 groups and Score 2 group versus Score 3, 4, 5, 6 groups (smoking duration); Score 1 group versus Score 6 group, Score 2 group versus Score 6 group, and Score 3 group versus Score 6 group (CRP); Score 1 group versus Score 2, 3, 4, 5, 6 groups, Score 2 group versus Score 4, 5, 6 groups, Score 3 group versus Score 4, 5, 6 groups, Score 4 group versus Score 5, 6 groups and Score 5 group versus Score 6 group (CPI)

### Physiological and radiological parameters

We investigated pulmonary function, ABGA results, and HRCT findings in IPF patients (Table 3). In Group 1, FVC (%) and DL_CO_ (%) were, respectively, 85.6 and 75.8 %, while in Group 6 the values were 55.5 and 31.9 %. ABGA also differed significantly among groups. Resting pulmonary oxygen tension (PaO_2_) was highest in Group 1, and higher GAP score was significantly associated with lower pulmonary oxygen tension (*p* < 0.001). In terms of radiological findings, the groups did not differ in any parameter other than reticular pattern.

**Table 3:** Initial physiologic and radiologic characteristics according to total GAP score

Variable	Total GAP score (*n* = 1,228)						*p*-value
	1 (*n* = 150)	2 (*n* = 208)	3 (*n* = 376)	4 (*n* = 317)	5 (*n* = 138)	6 (*n* = 39)	
Pulmonary function test							
FVC (%)	85.6 ± 13.4	81.9 ± 17.4	81.3 ± 17.2	71.4 ± 15.7	63.2 ± 15.1	55.5 ± 12.9	<0.001^a^
FEV_1_ (%)	93.1 ± 15.0	91.4 ± 21.2	92.5 ± 19.5	82.9 ± 16.8	74.4 ± 16.2	64.9 ± 14.9	<0.001^a^
TLC (%)	90.8 ± 19.7	84.8 ± 19.7	87.2 ± 18.5	80.2 ± 18.6	72.0 ± 15.6	67.1 ± 23.9	<0.001^a^
DL_CO_ (%)	75.8 ± 15.8	67.4 ± 17.1	67.1 ± 21.3	59.2 ± 19.9	41.7 ± 13.6	31.9 ± 11.1	<0.001^a^
Resting PaO_2_ mm Hg	90.5 ± 21.2	85.8 ± 19.6	82.5 ± 22.3	78.6 ± 18.7	74.5 ± 21.5	69.5 ± 13.7	<0.001^a^
Resting PaCO_2_ mm Hg	39.3 ± 8.2	39.2 ± 6.5	37.4 ± 7.8	36.5 ± 6.2	35.1 ± 6.9	36.7 ± 6.4	<0.001^a^
Radiologic finding							
Reticular pattern	108 (75.5)	144 (73.5)	214 (60.5)	185 (65.4)	82 (65.6)	22 (62.9)	0.008
Honeycombing change	105 (77.2)	141 (71.9)	282 (78.6)	241 (81.4)	110 (84.0)	31 (81.6)	0.101
Ground glass opacities	98 (68.5)	132 (68.4)	206 (58.9)	155 (58.5)	67 (56.3)	22 (68.8)	0.052
Nodular lesions	26 (20.3)	37 (19.9)	85 (25.5)	58 (23.6)	23 (21.9)	8 (25.8)	0.702

Note: Values in parentheses are percentages

*GAP* gender, age, and 2 lung physiology variables (FVC and DL_CO_), *FVC* forced vital capacity, *% pred* percentage of the predicted value, *FEV*_*1*_ forced expiratory volume, *TLC* total lung capacity, *DL*_*CO*_ diffusing capacity of the lung for carbon monoxide, *PaO*_*2*_ arterial oxygen tension, *PaCO*_*2*_ arterial carbon dioxide tension

^a^the following post hoc comparisons were significant at the *p* = 0.05 level; all other comparisons were non-significant: Score 1 group versus Score 4, 5, 6 groups, Score 2 group versus Score 4, 5, 6 groups, Score 3 group versus Score 4, 5, 6 groups and Score 4 group versus Score 5, 6 groups (FVC (%)); Score 1 group versus Score 4, 5, 6 groups, Score 2 group versus Score 4, 5, 6 groups, Score 3 group versus Score 4, 5, 6 groups and Score 4 group versus Score 5, 6 groups (FEV_1_ (%)); Score 1 group versus Score 4, 5, 6 groups, Score 2 group versus Score 5, 6 groups, Score 3 group versus Score 4, 5, 6 groups, and Score 4 group versus Score 5, 6 groups (TLC (%)); Score 1 group versus Score 2, 3, 4, 5, 6 groups, Score 2 group versus Score 4, 5, 6 groups, Score 3 group versus Score 4, 5, 6 groups, and Score 4 versus Score 5,6 groups (DL_CO_ (%)); Score 1 group versus Score 3, 4, 5, 6 groups, Score 2 group versus Score 4, 5, 6 groups, and Score 3 group versus Score 5, 6 groups (Resting PaO_2_); Score 1 group versus Score 4, 5 groups and Score 2 group versus Score 4, 5 groups (Resting PaCO_2_)

### Comorbidities and initial respiratory symptoms

Co-morbidities and initial presenting respiratory symptoms are shown in Additional file 1: Tables S1 and S2. The most common co-morbidities were past history of tuberculosis, diabetes mellitus, and hypertension; specifically, past history of tuberculosis was in 147 patients (12.0 %), diabetes mellitus in 234 (19.1 %), and hypertension in 271 (22.1 %). Furthermore, 74 patients (6.0 %) had lung cancer. These co-morbidities were not significantly different among groups. Fourteen patients (1.1 %) had a family history of IPF (data not shown). Cough, sputum, and hemoptysis were significantly more frequent at higher GAP scores (*p* = 0.004, *p* < 0.001, and *p* = 0.021, respectively). Although the proportion of patients who suffered dyspnoea of exertion increased as GAP score increased, this association was not statistically significant.

### Survival analysis on the basis of GAP score

**Fig. 2:**
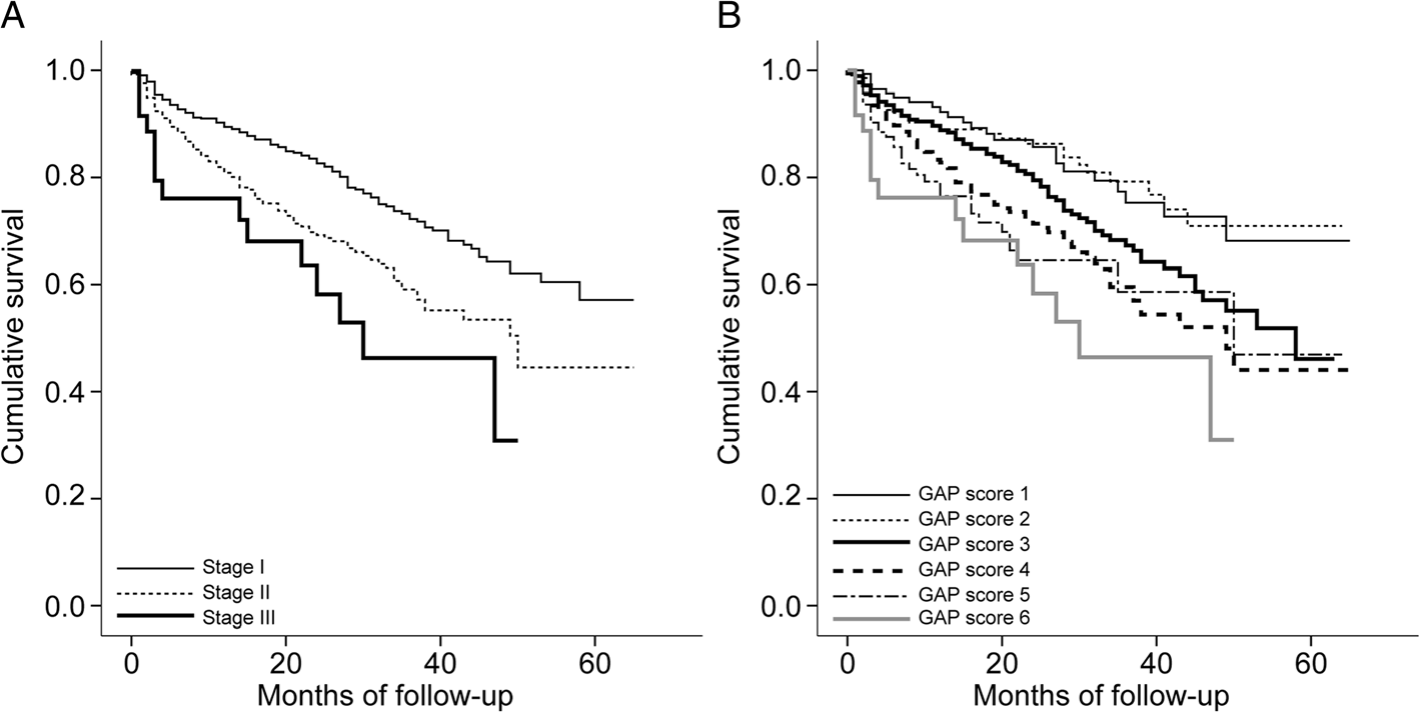
Kaplan-Meier estimates of survival of IPF patients based on (**a**) GAP stage, and (**b**) total GAP score. **a** Advanced GAP stages were significantly associated with poor prognosis (*p* < 0.001). **b** Cumulative survival in GAP score group 3 was significantly different from that in the other GAP score groups: GAP score 3 *vs.* GAP score 1, *p* = 0.043; GAP score 3 *vs.* GAP score 2, *p* = 0.039; GAP score 3 *vs.* GAP score 4, *p* = 0.043; GAP score 3 *vs.* GAP score 5, *p* = 0.032; GAP score 3 *vs.* GAP score 6, *p* = 0.003). Definition of abbreviations: GAP, gender, age, and two pulmonary physiology variables (FVC and DL_CO_)

All GAP variables showed significant association with prognosis except gender (G) (Table 4, Additional file 1: Table S3). The C-statistic values for the GAP stage at 1, 2, and 3 years were 0.59 (CI 0.537–0.638), 0.59 (CI 0.544–0.631), and 0.57 (CI 0.530–0.611), respectively. The GAP score showed a similar C statistic value with GAP stage. It was 0.61 (CI 0.556–0.653), 0.61 (CI 0.566–0.649), and 0.59 (0.549–0.627), respectively. Kaplan-Meier analysis was performed to compare survival among groups, as well as among GAP stages (Fig. 2a and b). Advanced GAP stage was associated with poor prognosis (*p* < 0.001). At GAP stages I and II (Groups 1–5), Group 3 differed significantly from all other groups in terms of cumulative survival (Group 3 *vs.* Group 1, *p* = 0.027; Group 3 *vs.* Group 2, *p* = 0.022; Group 3 *vs.* Group 4, *p* = 0.025; Group 3 *vs.* Group 5, *p* = 0.001). The causes of death are shown in Table 5. Respiratory failure (42.3 %) and infection (34.2 %) were the most common causes of death in study population.

**Table 4:** Survival analysis with Cox proportional hazard model

Variable	Univariate			Multivariate		
	HR	95 % CI	*p*-value	HR	95 % CI	*p*-value
Age	1.015	1.002–1.028	0.028	1.018	1.005–1.031	0.006
Sex (M/F)	1.184	0.890–1.575	0.245	1.264	0.949–1.684	0.109
FVC (%)	0.985	0.978–0.992	<0.001	0.986	0.979–0.993	<0.001
DL_CO_ (%)	0.987	0.981–0.993	<0.001	0.989	0.983–0.995	0.001

*FVC* forced vital capacity, *% pred* percentage of the predicted value, *DL*_*CO*,_ diffusing capacity of the lung for carbon monoxide

**Table 5:** Causes of death in the study population according to total GAP score

Variable	Total GAP score						Total
	1 (*n* = 11)	2 (*n* = 21)	3 (*n* = 60)	4 (*n* = 63)	5 (*n* = 28)	6 (*n* = 13)	
Respiratory failure	8 (72.7)	11 (52.4)	21 (35.0)	25 (39.7)	12 (42.9)	6 (46.2)	83
Infection	2 (18.2)	6 (28.6)	19 (31.7)	26 (41.3)	10 (35.7)	4 (30.8)	67
Heart failure			6 (1.0)	5 (7.9)	2 (7.1)	1 (7.7)	14
Lung cancer	1 (9.1)	4 (19.0)	9 (15.0)	5 (7.9)	2 (7.1)	1 (7.7)	22
Others^a^			5 (8.3)	2 (3.2)	2 (7.1)	1 (7.7)	10

Note: Values in parentheses are percentages

The cause of death was investigated in 196 mortality cases

*GAP* gender, age, and two lung physiology variables (FVC and DL_CO_)

^a^trauma or malignancy other than lung cancer

### Sub-analysis by GAP score

Table 6 shows the distribution of GAP points in each group in terms of predictive variables. Higher GAP scores were significantly associated with male predominance, aging, and poorer lung function, same as the original definition of the GAP model. Furthermore, gender, age, and DL_CO_ (%) differed significantly between Groups 2 and 3, and all four variables in the GAP model differed significantly between Groups 3 and 4.

**Table 6:** Distribution of GAP points by each predictor according to total GAP score

Variable	GAP Points	Total GAP score (*n* = 1,228)						*p*-value
		1 (*n* = 150)	2 (*n* = 208)	3 (*n* = 376)	4 (*n* = 317)	5 (*n* = 138)	6 (*n* = 39)	
Gender								<0.001
Female	0	59 (39.3)	94 (45.2)	95 (25.3)	48 (15.1)	15 (10.9)	4 (10.3)	
Male	1	91 (60.3)	114 (54.8)	281 (74.7)	269 (84.9)	123 (89.1)	35 (89.7)	
Age, yr								<0.001
≤60	0	121 (80.7)	76 (36.5)	45 (12.0)	15 (4.7)	6 (4.3)	-	
61–65	1	29 (19.3)	76 (36.5)	66 (17.6)	24 (7.6)	12 (8.7)	1 (2.6)	
>65	2	-	56 (26.9)	265 (70.5)	278 (87.7)	120 (87.0)	38 (97.4)	
Physiology								
FVC, % predicted								<0.001
>75	0	127 (84.7)	134 (64.4)	249 (66.2)	94 (29.7)	22 (15.9)	-	
50-75	1	23 (15.3)	71 (34.1)	121 (32.2)	211 (66.6)	92 (66.7)	22 (56.4)	
<50	2	-	3 (1.4)	6 (1.6)	12 (3.8)	24 (17.4)	17 (43.6)	
DL_CO_, % predicted								<0.001
>55	0	143 (95.3)	172 (82.7)	263 (69.9)	147 (46.4)	10 (7.2)	-	
36–55	1	7 (4.7)	35 (16.8)	108 (28.7)	156 (49.2)	81 (58.7)	12 (30.8)	
≤35	2	-	1 (0.5)	5 (1.3)	14 (4.4)	47 (34.1)	27 (69.2)	

Note: Values in parentheses are percentages

“Cannot perform” in DL_CO_ was not recorded in this study

Total GAP score 3 group was compared with each group 2 and group 4 by Bonferroni adjustment. The following post hoc comparisons were significant at the adjusted p value = 0.05; Score 3 group versus Score 2 group (Gender, age, and DL_CO_, % predicted); Score 3 group versus Score 4 group (Gender, age, FVC, % predicted and DL_CO_, %predicted)

*GAP* gender, age, and 2 lung physiology variables (FVC and DL_CO_)

## Discussion

The GAP model is simple to use in planning treatment or providing prognosis information to IPF patients. However, prognosis in relation to individual score groups have not been studied until now. This study attempted to undertake external validation of the GAP model in a relatively large cohort of IPF patients. Herein, we found that GAP score groups differed in terms of survival: in particular, survival in Group 3 patients differed from the other stage I groups, as well as the stage II groups.

For a long time, clinicians who care for IPF patients have been struggling to make accurate prognoses, because IPF is a heterogeneous disease that lacks a validated predictive model [11, 15]. Many previous researchers have aimed to find an ideal model for predicting clinical outcome in IPF patients [14, 16–22]. In 2001, for instance, King et al. [16] created an upgraded version of a previously existing clinical, radiological, and physiological scoring system, known as the “CRP system”, [17] to predict survival in IPF patients. This model took into account age, smoking status, clubbing of the fingertips, HRCT score, HRCT score for pulmonary hypertension score, TLC (%), and PaO_2_ at max exercise. However, it did not make clear that gender was significantly associated with mortality. Furthermore, it was too complex to use in a clinical setting, and cardio-pulmonary exercise testing was essential to calculating the score. Wells et al. [14] then proposed the composite physiological index (CPI), which used a combination of three factors to make a prediction—FVC (%), FEV_1_ (%), and DL_CO_ (%); these factors are determined using pulmonary function testing (PFT). Physicians could calculate CPI using PFT results only, rendering CT findings unnecessary in predicting prognosis. Besides these models, du Bois et al. [21] developed a predictive system that was based on IPF diagnostic criteria, and Richards et al. [22] used biomarkers to create another predictive model. However, these models have also been criticized because they are complicated to use or lack external validation.

Ley et al. developed the GAP model in 2012. Its straightforward nature has allowed the GAP index to be widely studied, [23–28] and it has been validated in the United States, Italy, and South Korea [12, 23]. In fact, the system showed robust predictive power in patients with chronic ILD (ILD-GAP model) and IPF related to occupational dust exposure [26, 28]. Furthermore, the model is more powerful and accurate when follow-up PFT results are taken into account, [26, 27] and it has been found that DL_CO_ can be replaced by HRCT fibrosis score in the GAP model (CT-GAP model) [25].

Interestingly, the duration of respiratory symptoms at diagnosis was longest in Group 1 and shortest in Group 6, although this was not a significant difference. This may be due to variations in individual perception of respiratory symptoms [29]. Hiwatari et al. [30] reported that IPF patients with mucous hypersecretion had significantly poor prognosis. In our study, the high score group showed sputum production significantly more often than score 1 or 2 group. This could mean that the patients with a higher GAP score could be more vulnerable to respiratory infection, which could be a cause of death. In our study, patients with a score over 3 showed a higher mortality rate due to infection than score 1 or 2 group. Variables related to smoking were significantly related to GAP score in this study; the proportion of ever-smokers, as well as smoking amount, were highest in Group 6. In other studies however, results have conflicted regarding the association between smoking and prognosis in IPF. Such results are easily influenced by gender, as well as the “healthy smoker effect” [16, 31]. In our study, smoking was not significantly associated with mortality in both univariate and multivariate analyses (Additional file 1: Table S3). Some investigations have shown that elevated CRP levels are related to poor prognosis [2, 32]. In the present study, CRP levels were highest in Group 6, and GAP score was significantly associated with CRP level (*p* < 0.001).

The most common cause of death in IPF patients is respiratory failure, which results from the progression of lung fibrosis, rather than comorbidities [3]. Furthermore, our study revealed no significant differences among the groups in terms of comorbidities. This suggests that mortality in IPF can be predicted, because the majority of mortalities are caused by the IPF itself.

In the present study, prognosis in Group 3 differed significantly from that in the other score groups, as shown using Kaplan-Meier analysis. This result suggests that the GAP score of 3 could be divided from the other stage I scores, thus creating a more refined prognostic system. Although the GAP model is simple to use and has proven effective in other chronic ILDs, the staging system amounts basically to a rough grouping of the GAP scores (stage I: 0–3 points, stage II: 4–5 points, and stage III: 6–8 points); the GAP stages I, II, and III were designed to have lowest 40 % risk, middle 40 % risk, and highest 20 % risk, respectively. In our study, Group 3 differed significantly from the other stage I groups, and from the stage II groups, in terms of all four predictive variables that contribute to GAP score; the only exception was FVC (%), which did not differ between Groups 2 and 3. Although the mean value of lung function results was similar, age and gender composition were significantly different between Group 2 and 3. Ley et al. [12] mentioned that one of the limitations of the GAP model is its overestimation of risk in lower-risk groups, and this may be the reason for the lack of significant difference in FVC (%) mentioned between Group 2 and 3. Although the mean value of CPI significantly increased as GAP score increased, the difference of CPI between Groups 2 and 3 was not significant in our study, unlike GAP score. This might be explained by a difference in study design between the GAP model and CPI: GAP uses more clinical data in its model, such as age and gender, while CPI was created using only PFT results [12, 14].

Our study did have some limitations. Firstly, patients were diagnosed using the 2002 ATS/ERS guidelines, which place more importance on surgical lung biopsy results than do the 2011 updated guidelines. Also in this study, the HRCT findings were not quantified as scores, or classified according to updated guidelines. In addition, in radiologic findings, traction bronchiectasis was not investigated. However, Ley et al. [12] created the GAP model using a derivation cohort and validation cohort that had been diagnosed between 2000 and 2010. Additionally, Kim et al. [23] demonstrated that the GAP model was effective (except in predicting the 3-year risk of death) in Korean IPF patients who had been diagnosed between 2005 and 2009. Another limitation is that Groups 0 and 7 were excluded from the study because they contained much fewer patients than the other score groups. In fact, patients in Group 0 (all women, never smokers) differed significantly from the other score groups in terms of baseline characteristics. Furthermore, no patients were enrolled who had a GAP score of 8, which requires the inclusion of an “unable to perform” category in DL_CO_ measurement. We also excluded patients who had not undergone PFT that included DL_CO_. This considerable number of excluded groups may have led to selection bias. Finally, the Korean ILD group did not investigate the radiologic scoring of fibrosis, dyspnea scale, and pulmonary artery hypertension, which could have provided more information on prognosis in IPF patients.

## Conclusion

In summary, this study was designed as a national validation study to evaluate GAP scores in relation to the prognosis of patients with IPF. On the basis of our study results, we suggest that Group 3 could be separated from other GAP stage I patients and that reporting this score separately would improve mortality prediction.

## Additional file

Additional file 1: Table S1. Comorbidities of idiopathic pulmonary fibrosis patients according to GAP score. **Table S2.** Initial presenting symptoms of study population. **Table S3.** Survival analysis with Cox proportional hazard model including age, sex, FVC (%), DL_CO_ (%), and smoking. (DOCX 23 kb)

## Abbreviation

% pred: Percentage of the predicted value
ABGA: Arterial blood gas analysis
CPI: Composite physiologic index
DL_CO_: Diffusing capacity of the lung for carbon monoxide
FEV_1_: Forced expiratory volume
FVC: Forced vital capacity
GAP: Gender, age, and 2 lung physiology variables (FVC and DL_CO_)
HRCT: High-resolution computed tomography
ILD: Interstitial lung disease
IPF: Idiopathic pulmonary fibrosis
PaCO_2_: Arterial carbon dioxide tension
PaO_2_: Arterial oxygen tension
PFT: Pulmonary function test
TLC: Total lung capacity
