# Supplementary material for: Predicting survival of patients with idiopathic pulmonary fibrosis using GAP score: a nationwide cohort study
Source: Respir Res. 2016 Oct 18;17:131. doi: 10.1186/s12931-016-0454-0 (PMC5069824; doi:10.1186/s12931-016-0454-0)
Supplement: Additional file 1: Table S1. — Comorbidities of idiopathic pulmonary fibrosis patients according to GAP score. Table S2. Initial presenting symptoms of study population. Table S3. Survival analysis with Cox proportional hazard model including age, sex, FVC (%), DLCO (%), and smoking. (DOCX 23 kb) [file 12931_2016_454_MOESM1_ESM.docx]

**<Additional file>**

Title: Predicting survival of patients with idiopathic pulmonary fibrosis using GAP score: A nationwide cohort study

Author list: *S. H. Lee, S.Y. Kim, D. S. Kim, Y .W. Kim, M. P. Chung, S. T. Uh, C. S. Park, S. H. Jeong, Y. B. Park, H. L. Lee, J. W. Shin, E. J. Lee, J. H. Lee, Y. Jegal, H. K. Lee, Y. H. Kim, J. W. Song, S. W. Park, M. S. Park*

**Table S1**. Comorbidities of idiopathic pulmonary fibrosis patients according to GAP score

| Variable | Total GAP score (n=1,228) | | | | | | p-value |
| --- | --- | --- | --- | --- | --- | --- | --- |
|  | 1 (n=150) | 2 (n=208) | 3 (n=376) | 4 (n=317) | 5 (n=138) | 6 (n=39) |  |
| Past history of Tuberculosis | 20 (13.3) | 31 (15.0) | 45 (12.0) | 32 (10.1) | 14 (10.1) | 5 (13.2) | 0.611 |
| Diabetes mellitus | 28 (18.7) | 40 (19.2) | 76 (20.2) | 52 (16.4) | 30 (21.7) | 8 (20.5) | 0.778 |
| Hypertension | 26 (17.3) | 51 (24.5) | 89 (23.7) | 62 (19.6) | 35 (25.4) | 8 (20.5) | 0.377 |
| Cardiovascular disease | 6 (4.0) | 9 (4.3) | 32 (8.5) | 19 (6.0) | 13 (9.4) | 3 (7.7) | 0.182 |
| Cerebrovascular disease | 3 (2.0) | 1 (0.5) | 7 (1.9) | 11 (3.5) | 5 (3.6) | 2 (5.1) | 0.178 |
| Liver disease | 1 (0.7) | 7 (3.4) | 4 (1.1) | 5 (1.6) | 4 (2.9) | 0 (0.0) | 0.208 |
| Allergic disease | 3 (2.0) | 7 (3.4) | 5 (1.3) | 1 (0.3) | 1 (0.7) | 1 (2.6) | 0.098 |
| Renal disease | 1 (0.7) | 1 (0.5) | 3 (0.8) | 8 (2.5) | 5 (3.6) | 1 (2.6) | 0.004 |
| Lung cancer | 8 (5.3) | 12 (5.8) | 25 (6.6) | 17 (5.4) | 10 (7.2) | 2 (5.1) | 0.956 |
| Other malignancy | 2 (1.3) | 8 (3.8) | 17 (4.5) | 17 (5.4) | 9 (6.5) | 4 (10.3) | 0.142 |

Note: Values in parentheses are percentages.

GAP = gender, age, and 2 lung physiology variables (FVC and DLco)

**Table S2.** Initial presenting symptoms of study population

| Variable | Total GAP score (n=1,228) | | | | | | p-value |
| --- | --- | --- | --- | --- | --- | --- | --- |
|  | 1 (n=150) | 2 (n=208) | 3 (n=376) | 4 (n=317) | 5 (n=138) | 6 (n=39) |  |
| Dyspnoea of exertion | 94 (73.4) | 143 (76.9) | 265 (82.0) | 218 (82.9) | 93 (83.8) | 29 (85.3) | 0.138 |
| Cough | 83 (66.9) | 117 (66.9) | 222 (72.1) | 200 (81.3) | 78 (79.6) | 25 (78.1) | 0.004 |
| Sputum | 46 (39.0) | 55 (35.3) | 117 (47.0) | 118 (59.3) | 54 (64.3) | 18 (60.0) | <0.001 |
| Hemoptysis | 1 (1.1) | 0 (0.0) | 11 (5.4) | 10 (6.9) | 3 (4.9) | 2 (10.0) | 0.021 |
| Chest pain | 10 (10.1) | 17 (12.3) | 21 (10.2) | 16 (10.8) | 8 (12.5) | 5 (23.8) | 0.563 |
| Asymptom | 11 (36.7) | 14 (37.8) | 22(25.3) | 6 (10.3) | 3 (10.0) | 0 (0.0) | 0.002 |

Note: Values in parentheses are percentages.

GAP, gender, age, and 2 lung physiology variables (FVC and DL_CO_)

**Table S3**. Survival analysis with Cox proportional hazard model including age, sex, FVC (%), DL_CO_ (%), and smoking

| Variable | Univariate | | | Multivariate | | |
| --- | --- | --- | --- | --- | --- | --- |
|  | HR | 95% CI | p-value | HR | 95% CI | p-value |
| Age | 1.015 | 1.002-1.028 | 0.028 | 1.015 | 0.998-1.031 | 0.082 |
| Sex (M/F) | 1.184 | 0.890-1.575 | 0.245 | 1.261 | 0.614-2.588 | 0.527 |
| FVC (%) | 0.985 | 0.978-0.992 | <0.001 | 0.986 | 0.977-0.995 | 0.003 |
| DL_CO_ (%) | 0.987 | 0.981-0.993 | <0.001 | 0.992 | 0.985-1.000 | 0.043 |
| Smoking | 0.996 | 0.989-1.004 | 0.375 | 0.996 | 0.987-1.004 | 0.324 |

FVC, forced vital capacity; % pred, percentage of the predicted value; DL_CO,_ diffusing capacity of the lung for carbon monoxide
